# Beyond linguistic competence: shifting classroom environments and motivational paths to foster self-regulated oral presentations—a theoretical framework development

**DOI:** 10.3389/fpsyg.2026.1901333

**Published:** 2026-09-07

**Authors:** Jingran Wang, Chang'an Wu, Shuang Peng

**Affiliations:** School of Chinese Language and Literature, Northeast Normal University, Changchun, China

**Keywords:** academic oral presentation, classroom environment, college English, Confucian heritage culture, EFL, motivational beliefs, self-regulated learning

## Abstract

Academic oral presentations have become a defining competency of higher education in the era of internationalized scholarship, demanding not only linguistic accuracy but also sophisticated self-regulated learning capacities. However, despite the growing recognition of self-regulated learning as a critical mechanism in second/foreign language acquisition, educational psychology research has been disproportionately preoccupied with receptive skills such as reading, while productive oral skills—particularly the high-stakes, performance-laden context of academic oral presentations—have remained theoretically underdeveloped. Moreover, while individual differences in motivation and anxiety have received substantial attention, the role of the immediate classroom environment in shaping students’ self-regulated learning has remained peripheral, and the mediating mechanisms through which environmental factors translate into strategic behavior have not been adequately theorized. To bridge these gaps, this paper develops a theoretical framework articulating how Chinese college English learners’ perceived classroom environment shapes their self-regulated learning strategy use in academic oral presentations through motivational beliefs. Drawing on the social-cognitive perspective and on motivational theories of expectancy-value, self-efficacy, and achievement goals, the framework conceptualizes classroom environment along two dimensions—instructional practices (presentation task design, process-oriented teacher support, study autonomy) and social support (teacher and peer social support)—and posits five motivational beliefs (utility value, intrinsic interest, self-efficacy, mastery goals, and performance goals) as mediators. We propose that (a) presentation task design and process-oriented teacher support exert direct effects on strategy use, while study autonomy operates indirectly via intrinsic interest; (b) teacher social support exerts broad multi-path indirect effects via several motivational beliefs, whereas peer social support, owing to the audience-evaluator duality unique to oral presentations, operates through a reconfigured set of motivational pathways centered on self-efficacy and utility value; and (c) the Confucian Heritage Culture context of Chinese higher education moderates these pathways in distinctive ways. This reconfigured framework generates testable propositions for future empirical inquiry, advances self-regulated learning theory into the productive oral domain, and informs the design of motivationally responsive pedagogy.

## 1 Introduction

In an era of accelerating globalization and internationalized scholarship, the ability to deliver effective academic oral presentations (AOPs) in English has become a defining competency for university students worldwide. Whether in seminar discussions, conference talks, thesis defenses, or career-oriented professional pitches, AOPs require students to integrate linguistic accuracy, content organization, audience awareness, multimodal communication, and—crucially—a high degree of self-regulation under time-bound and socially evaluative conditions (Mercer, 2019). For Chinese college English learners, who typically transition from an examination-intensive secondary school system (Chen et al., 2023; Zhong, 2006) into a higher education context that ostensibly emphasizes autonomy and academic inquiry, the AOP often represents not merely a linguistic challenge but a comprehensive self-regulatory ordeal. Anxiety, fragmented planning, poor in-performance monitoring, and weak post-performance reflection are widely reported in pedagogical accounts but inadequately theorized in the research literature (Dao and Sato, 2021; Payant and Zuniga, 2022).

Self-regulated learning (SRL)—broadly conceptualized as an active, constructive process whereby learners proactively set goals, monitor their progress, and systematically control their cognition, motivation, and behavior to accomplish complex tasks (Pintrich, 2000)—has emerged as a foundational pillar of scholastic success. In the context of academic oral presentations (AOPs), SRL specifically refers to learners’ self-directed orchestration of preparation planning, affective regulation, multimodal delivery monitoring, and post-performance reflection. Within the social-cognitive tradition, SRL is conceptualized as emerging from the dynamic interplay between individual characteristics and environmental conditions (Pintrich and Zusho, 2002; Winne, 2017). Yet the bulk of research on L2 SRL—and on classroom factors influencing SRL—has been disproportionately focused on receptive skills, particularly reading, and to a lesser extent on writing (Lau, 2012a, 2012b; Teng and Zhang, 2016; Zhang et al., 2014). The productive oral skills domain, and especially the high-stakes performance context of AOPs, has remained theoretically underdeveloped.

We identify three critical gaps in the current literature that motivate the present theoretical contribution.

The skill bias gap: Foundational SRL studies in L2 contexts have predominantly examined receptive skills, particularly reading comprehension (Chen et al., 2021, 2023; Lau, 2019, 2020; Zhang et al., 2014) or written composition (Teng and Zhang, 2016). Although these studies have substantially advanced our understanding of SRL in receptive and asynchronous productive skills, their findings cannot be straightforwardly extrapolated to the oral presentation context. In this specific domain, temporal immediacy, multimodal communication, and live audience evaluation impose qualitatively distinct cognitive and affective demands on the learner (Baralt et al., 2016; Ke, 2025). The few existing studies on L2 oral SRL have generally focused on conversational or interactional speaking (Dörnyei and Kormos, 2000; Lambert et al., 2017; Lambert et al., 2023), leaving the structured, monologic, and academically situated AOP largely unexamined.

The environmental neglect gap: Despite robust theoretical recognition that SRL is environmentally embedded (Bronfenbrenner and Morris, 1998; Skinner et al., 2022), much of the L2 SRL literature continues to emphasize individual learner characteristics—such as motivation, personality, and prior achievement—at the expense of systematic attention to the immediate, malleable classroom environment (Chen et al., 2023; Teng and Zhang, 2016). When environmental factors are considered, they are often treated as homogeneous background variables rather than as a structured ecology with differentiated dimensions, failing to separate instructional practices from social support networks (Patrick et al., 2007; Roorda et al., 2017). This omission is particularly problematic for AOP pedagogy, where classroom design choices—how presentation tasks are structured, how teachers scaffold the preparation process, and how peers function as both collaborators and audience—exert powerful but uncharted influences on students’ SRL development.

The mechanism gap: regarding the mechanism gap, social-cognitive motivational theories posit that the relationship between the classroom environment and student engagement is mediated by learners’ motivational beliefs (Ames, 1992; Eccles and Wigfield, 2002; Pintrich, 2004). As recent bibliometric assessments of educational psychology emphasize, issues surrounding student motivation, self-regulation, and learning strategies consistently constitute the field’s most impactful and heavily cited domains (Hassan et al., 2024). Empirical evidence in general education and first-language reading contexts has confirmed such mediation (Lau, 2012a, 2012b; Patrick et al., 2007; Song et al., 2015).

To bridge these gaps, the overarching objective of this paper is to develop an integrative, social-cognitive theoretical framework that conceptualizes how perceived classroom environment dimensions (instructional practices and social support) drive Chinese college English learners’ self-regulated learning (SRL) strategy deployment in academic oral presentations (AOPs) through five core motivational beliefs. Specifically, this theoretical contribution aims to: (1) extend the social-cognitive SRL paradigm beyond traditional receptive domains into real-time, high-stakes oral production; (2) disentangle the direct scaffolding and motivationally mediated pathways through which instructional practices and social support translate into strategic action; and (3) elucidate how the Confucian Heritage Culture (CHC) setting of Chinese higher education moderates these relational pathways.

In doing so, this paper carries dual theoretical and practical significance. Theoretically, it opens the motivational “black box” linking malleable classroom ecologies to multidimensional oral SRL behaviors while reconfiguring peer support around the audience-evaluator duality unique to oral presentations. Pedagogically, it provides university educators with actionable principles to move beyond linguistic drill-and-practice by structuring motivationally responsive learning environments and scaffolded presentation tasks.

## 2 Theoretical foundations and literature review

### 2.1 Self-regulated learning: a social-cognitive anchor

#### 2.1.1 A social-cognitive anchor

SRL has been theorized within multiple traditions, including phase models (Zimmerman and Moylan, 2009), information-processing approaches (Winne, 2017), and social-cognitive frameworks (Pintrich, 2000, 2004). Following Pintrich (2000), we adopt the social-cognitive perspective, which conceptualizes SRL as an active, goal-directed process whereby learners monitor, regulate, and control their cognition, motivation, and behavior in the service of learning. Critically, this perspective treats SRL not as a stable trait but as an emergent property of the interaction between learner characteristics and environmental affordances (Paris and Paris, 2001; Pintrich and Zusho, 2002; Skinner et al., 2022). It is this interactional emphasis that makes the social-cognitive perspective particularly suitable for theorizing classroom-level influences.

Building on Pintrich’s foundation, Teng and Zhang (2016) developed an influential L2 SRL framework that identifies four interrelated components—cognitive, metacognitive, motivational, and social-behavioral regulation—each corresponding to a class of strategies. Cognitive strategies are deployed to process information and accomplish tasks; metacognitive strategies monitor and regulate cognition; motivation-regulation strategies sustain effort and affect; and social-behavioral strategies manage the learner’s interaction with the environment (Oxford, 2013; Teng and Zhang, 2016). Consistent with Chen et al. (2023), we focus on cognitive, metacognitive, and motivation regulation as the core SRL components, given their centrality to deep processing and long-term competence development.

#### 2.1.2 The distinctive demands of the academic oral presentation context

Although the social-cognitive SRL framework is in principle applicable across learning domains, its specific operationalization must be tailored to the demands of the focal context (Hiver et al., 2021; Teng and Zhang, 2016). The AOP context imposes at least three qualitatively distinctive demands that differentiate it from receptive skills such as reading.

First, temporal immediacy and online cognitive multitasking. Unlike reading, where learners can pause, reread, and reflect at will, AOPs unfold in real time under continuous audience scrutiny. In this transient communication context, the luxury of offline recursive processing is entirely absent, meaning that self-regulation must operate dynamically under severe cognitive and time constraints (Dörnyei and Kormos, 2000; Johnson and Abdi Tabari, 2022). Presenters must simultaneously manage conceptual planning, grammatical formulation, and speech articulation (Asadnia and Atai, 2022). At the same time, they must track audience reactions and manipulate visual slides in real time. This synchronous cognitive load substantially elevates the demand on metacognitive monitoring and online emotion regulation. Indeed, while foundational research in educational technology underscores that lecture-based multitasking severely taxes cognitive capacity and impairs individual retention (McVaugh and Robinson, 2022), the academic oral presentation context further amplifies this executive strain, meaning that strategic corrections and coping mechanisms must be deployed within fractions of a second (Dörnyei and Kormos, 2000; Johnson and Abdi Tabari, 2022).

Second, high affective load and emotional fluctuations. Oral academic performance is inherently linked to elevated vulnerability and severe foreign language speaking anxiety (FLSA), particularly when delivered in evaluative higher education contexts (Dao and Sato, 2021; Payant and Zuniga, 2022; Coskun, 2017). Effective AOP self-regulation thus requires highly sophisticated emotion-management and motivation-regulation strategies—a domain that has been comparatively peripheral in the SRL literature on receptive skills (Teng and Zhang, 2016). Drawing on contemporary principles of Positive Psychology in SLA, language learning emotions are conceptualized not as static states, but as dynamic, interacting systems where negative anxiety operates in tandem with positive Foreign Language Enjoyment (FLE) (Bashori et al., 2021; Zhang, 2024). Consequently, self-regulation in an AOP classroom becomes an active psychological negotiation, wherein learners must deploy internal strategies to maintain emotional resilience, exploit social buffers, and manage severe face-threats to prevent performance impairment and communication withdrawal (Dörnyei and Kormos, 2000; Gao and Tay, 2023).

Third, multimodal communication and gestural orchestration. AOPs are inherently multimodal communicative events that extend far beyond the linguistic surface of verbal output. Presenters must integrate and coordinate intonation, speech pacing, gaze direction, posture, and coverbal body gestures alongside integrated visual aids (Baralt et al., 2016; Ke, 2025). Classroom gesture research demonstrates that spontaneous manual movements are closely synchronized with the speaker’s conceptual ideas and serve critical discourse functions (Ma and Jin, 2022). For instance, rhythmic beats gestures function as visual punctuations that signal discourse prominence and assist in speech segmentation, while representational metaphorics externalize abstract scientific concepts into tangible, imagistic spatial structures, effectively reducing the working memory load for both the speaker and the listener (Ma and Jin, 2022). Strategic regulation in the AOP context must therefore encompass the intentional orchestration of these multimodal semiotic channels to construct cohesive academic discourse and successfully sustain remote audience attention (Asadnia and Atai, 2022; Ma and Jin, 2022).

#### 2.1.3 SRL strategies in the academic oral presentation context

We propose the following operationalization of SRL strategies for the AOP context, drawing on and adapting prior frameworks (Pintrich, 1991; Teng and Zhang, 2016; Zhang et al., 2014).

Cognitive strategies include information selection and integration from source materials; logical structuring of the presentation argument; drafting of speech outlines or scripts; preparation for potential Q&A; and the strategic use of examples, evidence, and rhetorical devices to support claims. These strategies parallel the deep-processing functions identified in reading SRL (Chen et al., 2023; Zhang et al., 2014) but are oriented toward production rather than comprehension.

Metacognitive strategies include goal-setting and pre-presentation planning (audience analysis, time allocation); rehearsal monitoring (recognizing weak spots, adjusting pace or content); in-performance monitoring (gauging audience response, recovering from disruptions); and post-presentation reflection (evaluating performance against goals, identifying areas for future improvement). These strategies are crucial for moving from one-off task completion to cumulative skill development (Paris and Paris, 2001; Zimmerman and Moylan, 2009).

Motivation-regulation strategies include anxiety management (e.g., breathing techniques, cognitive reappraisal); self-instruction and self-encouragement (e.g., internal pep-talks before going on stage); interest enhancement (e.g., choosing topics personally meaningful to the speaker); and persistence regulation (e.g., maintaining effort across the often-prolonged preparation phase). The salience of this dimension is, as noted above, particularly high in the AOP context.

### 2.2 The proximal classroom environment: a two-dimensional lens

#### 2.2.1 Bioecological grounding

That classroom environment matters for student learning is a near-axiomatic claim in contemporary educational psychology. Bronfenbrenner and Morris’s (1998) bioecological model identifies the classroom as a critical proximal microsystem within which developmental processes unfold. More recent elaborations (Skinner et al., 2022) emphasize that classroom-level factors—teaching practices, interpersonal relationships, peer cultures—are particularly proximal to students’ daily learning experiences and therefore particularly potent in shaping motivation and strategic behavior.

Yet, as Lau (2012a, 2012b) and Dignath and Veenman (2021) have observed, research on classroom effects on SRL has often suffered from two limitations: a tendency to treat the classroom as a unitary construct, obscuring within-class heterogeneity in instructional and relational dynamics; and a reliance on small-scale qualitative methods, limiting generalizability. To address these limitations theoretically, the present framework adopts a two-dimensional conceptualization of the classroom environment that distinguishes instructional practices from social support (Patrick et al., 2007; Wentzel, 2022). These two dimensions are conceptually complementary: instructional practices specify what teachers deliberately do to scaffold strategic learning, while social support specifies the relational quality that energizes engagement (Skinner et al., 2022).

#### 2.2.2 Dimension 1: Instructional practices

In educational psychology and instructional methodology, instructional practices are formally defined as the intentional pedagogical designs, task structures, and procedural scaffolding through which educators deliberately guide and facilitate student learning processes (Perry, 1998; Dignath and Veenman, 2021). Drawing on Lau's (2012a, 2012b) Chinese SRL-based Reading Instruction framework—which integrates Perry’s (1998) classroom observation tool with Ames’s (1992) TARGET model—and on Chen et al.’s (2026) recent two-factor reformulation of this construct in mainland Chinese contexts, we identify three subdimensions particularly salient for AOP pedagogy.

Presentation task design. This subdimension encompasses the cognitive and communicative qualities of the AOP tasks teachers assign, including their authenticity (e.g., simulating real academic genres such as conference talks or thesis defenses), challenge level (e.g., requiring synthesis of multiple sources), and meaningfulness (e.g., connection to students’ research interests or career trajectories). Well-designed presentation tasks function as authentic contexts for SRL deployment, providing the substrate against which students can develop and refine strategic competence (Dignath and Büttner, 2018; Lau, 2019).

Process-oriented teacher support. Beyond task design, teachers can support AOP development by offering scaffolded guidance across the preparation cycle: helping students select topics, organize content, design visual aids, and rehearse delivery; modeling effective strategies (e.g., think-alouds during script revision); and providing formative feedback that targets both linguistic and rhetorical dimensions (Dignath and Veenman, 2021; Spruce and Bol, 2015). This subdimension corresponds to what Chen et al. (2026) identified as part of the “instructional task” factor in their two-factor analysis of SRL-based instruction.

Study autonomy. This subdimension captures the degree to which students are afforded choice, self-direction, and ownership in the AOP process—including, for example, opportunities to select topics, formats, and evaluation criteria; to engage in self-assessment and peer-assessment; and to negotiate task parameters with the teacher (Ryan and Deci, 2009). Study autonomy has been advocated in Western pedagogical traditions (Perry, 1998; Perry et al., 2023) and aligns with the self-determination principle that autonomy support nurtures intrinsic motivation. While Western research demonstrates that autonomy support directly enhances student engagement (Perry, 1998; Ryan and Deci, 2009), empirical findings in Confucian Heritage Culture (CHC) contexts are noticeably divergent. For instance, comparative studies by Lau and Chen (2013) between Beijing and Hong Kong learners revealed that perceived autonomy did not directly translate into reading strategy gains unless mediated by intrinsic interest. Similarly, Schweisfurth (2015) documented that open-ended learner autonomy often creates cognitive dissonance in examination-driven Chinese classrooms unless accompanied by structured teacher scaffolding.

#### 2.2.3 Dimension 2: Social support

Complementing instructional practices, social support denotes the perceived quality of interpersonal relationships within the classroom microsystem, manifested in both academic assistance (instrumental help) and socio-emotional care (encouragement and anxiety buffering) from educators and peers (Patrick et al., 2007; Wentzel et al., 2010). Two sources of social support are particularly salient: teachers and peers.

Teacher social support. This refers to students’ perceptions that the teacher cares about them personally and provides assistance when needed (Johnson et al., 1983; Patrick et al., 2007). In the AOP context, teacher social support may manifest in respectful engagement with students’ presentation ideas, sensitivity to performance anxiety, and the provision of reassurance and personalized encouragement throughout the preparation and performance cycle. The literature on teacher-student relationships and academic motivation suggests that teacher support is a particularly potent predictor of adolescent engagement (Roorda et al., 2017; Tao et al., 2022), with effects often channeled through students’ motivational beliefs (Wentzel, 1999, 2022).

Peer social support. This refers to students’ perceptions that classmates care about and support them, both academically and emotionally (Wentzel et al., 2010). Importantly, in the AOP context, peers occupy a structural role qualitatively different from their role in reading or written assignment contexts: they are simultaneously fellow learners, audience members, and—increasingly—peer feedback providers (He and Jin, 2026; Liu and Hansen, 2002). This dual role has significant implications for how peer support operates as an environmental input. Recent empirical investigations into L2 peer review delineate a clear structural divergence between written and oral tasks. Whereas peer interaction in writing is largely asynchronous and evaluative (Liu and Edwards, 2018; Zhu and To, 2022), oral presentations position peers in a live, synchronous dual role as both evaluators and immediate audience members (He and Jin, 2026; Ke, 2025). Empirical evidence from Wu and Schunn (2023) indicates that affective peer reassurance serves as an essential psychological buffer against live face-threats during public speaking.

### 2.3 The motivational mediating system

#### 2.3.1 Three theoretical traditions

Within the social-cognitive paradigm, motivational beliefs are defined as learners’ subjective cognitive and affective appraisals regarding their perceived competence, subjective task values, and goal orientations that energize, direct, and sustain goal-oriented effort (Eccles and Wigfield, 2002; Pintrich, 2004). In our framework, these beliefs function as the internal psychological engine converting external environmental affordances into concrete self-regulated actions. Expectancy-value theory (Eccles and Wigfield, 2002) differentiates subjective task value into multiple components. We focus on two especially relevant to the AOP context: utility value (how a task relates to current and future goals, such as academic advancement or career aspirations) and intrinsic value, sometimes operationalized as interest (the enjoyment derived from engaging in the task itself).

Self-efficacy theory (Bandura, 1997) posits that beliefs in one’s capability to execute a specific task strongly influence the effort, persistence, and strategy use that one invests. In the AOP context, self-efficacy is closely intertwined with performance anxiety, since low confidence in one’s presentation ability tends to amplify both anticipatory and in-performance anxiety (Dao and Sato, 2021).

Achievement goal theory (Ames, 1992) distinguishes mastery goals (oriented toward learning, improvement, and deep understanding) from performance goals (oriented toward demonstrating competence relative to others or obtaining external rewards). Although mastery goals are generally associated with adaptive learning patterns, recent evidence from CHC contexts suggests that performance goals can also be functionally adaptive under examination-intensive conditions (Chen et al., 2023).

#### 2.3.2 Five motivational beliefs in the AOP context

Integrating these three traditions, we identify five motivational beliefs as the focal mediators in our framework.

1. Utility value: Students’ perceptions of the usefulness of AOP skills for academic success, scholarly communication, and career trajectories. In a context where Chinese college English learners are increasingly expected to communicate research findings in international forums, the utility value of AOP competence is likely to be salient (Zhong, 2006).
2. Intrinsic interest: Students’ enjoyment of and engagement with the act of presenting itself—the intellectual pleasure of articulating ideas, the satisfaction of audience connection, the creative dimension of crafting a presentation. Intrinsic interest has been theorized as a particularly powerful driver of deep, sustained engagement (Ryan and Deci, 2009).
3. Self-efficacy: Students’ confidence in their ability to plan, prepare, deliver, and adaptively manage an academic oral presentation. Given the high affective load of AOPs, self-efficacy is likely to be especially consequential for both strategy use and anxiety management.
4. Mastery goals: Students’ orientation toward learning and improving their AOP competence as an end in itself, rather than as a means to external validation.
5. Performance goals: Students’ orientation toward demonstrating AOP competence relative to peers or obtaining favorable evaluations from teachers.

Together, these five beliefs constitute the motivational engine through which classroom environment is hypothesized to influence SRL strategy use.

## 3 Proposed framework and testable hypotheses

**Figure 1:**
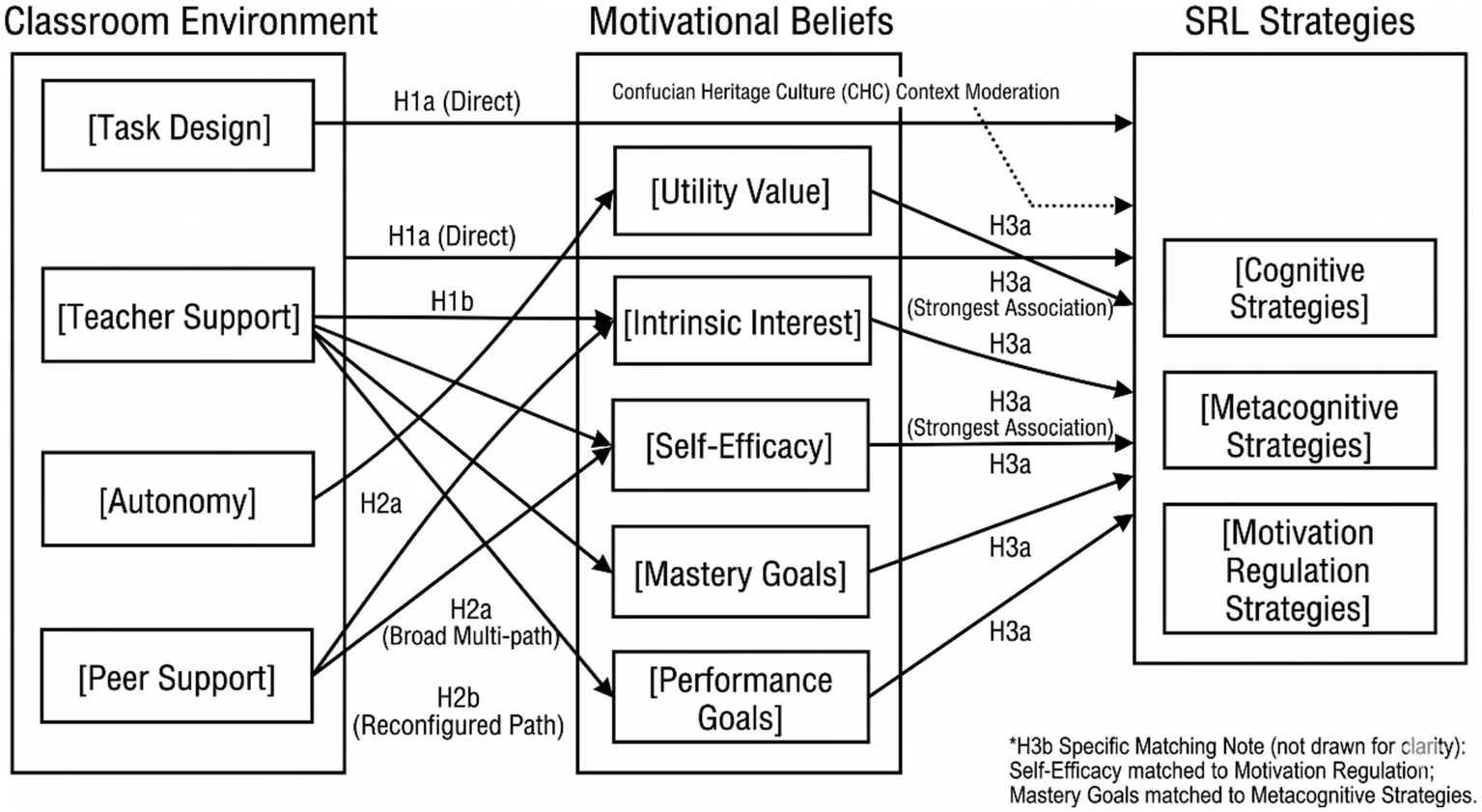
The conceptual path model of classroom environment, motivational beliefs, and self-regulated learning (SRL) strategies in academic oral presentations (AOPs).

We now integrate the components into a unified theoretical framework. The framework posits that the two-dimensional classroom environment (instructional practices and social support) influences SRL strategy use in college English AOPs primarily through the five motivational beliefs, with the strength and pattern of mediation varying systematically across environmental and motivational dimensions. We organize our propositions into three distinct hypothesis clusters (see Figure 1).

### 3.1 Hypothesis cluster 1: Differential effects of instructional practices

Drawing on Chen et al.'s (2026) recent findings in the high school EFL reading context and extending these to the AOP domain, we propose that the subdimensions of instructional practices operate through distinct pathways.

Theoretical Derivation for Instructional Practices (H1a & H1b):

To delineate the mechanisms through which instructional practices act upon strategic learning, we untangle their pathways based on Self-Determination Theory (SDT; Ryan and Deci, 2009) and Cognitive Scaffolding paradigms (Dignath and Büttner, 2018). In our framework, study autonomy support represents a crucial distal environmental affordance that directly satisfies students’ basic psychological needs for competence and self-direction. When learners are granted ownership over topic selection and presentation formats, this autonomy satisfaction directly nurtures their intrinsic task value, transforming into heightened intrinsic interest, which subsequently triggers spontaneous cognitive and metacognitive strategy enactment (Ryan and Deci, 2009; Chen et al., 2026). Conversely, presentation task design and process-oriented teacher support operate as an immediate instructional scaffold (Dignath and Veenman, 2021). Academic oral presentations represent highly structured and complex communicative genres. Consequently, explicit process-oriented teacher guidance lowers the initial executive threshold for strategy deployment. By providing concrete procedural templates, this instructional scaffolding enables students to enact cognitive and metacognitive strategies directly, without requiring prior motivational activation (Dignath and Büttner, 2018; Chen et al., 2026).

*H1a:* Presentation task design and process-oriented teacher support exert direct positive effects on SRL strategy use in AOPs. The rationale is that well-designed, scaffolded tasks provide concrete templates and opportunities for strategy deployment, allowing students to enact SRL even in the absence of strong motivational mediation (Dignath and Büttner, 2018; Lau, 2019). This proposition is consistent with Chen et al.'s (2026) finding that the “instructional task” factor exerted only a direct effect on SRL in the reading context, though we anticipate a somewhat stronger effect in the AOP context owing to the greater task complexity and the consequent greater payoff of structured support.

*H1b:* Study autonomy exerts an indirect effect on SRL strategy use primarily via intrinsic interest. The rationale here is rooted in self-determination theory (Ryan and Deci, 2009): autonomy support satisfies the basic psychological need for self-direction, which in turn fosters intrinsic interest, which then activates deeper engagement with SRL strategies. This proposition mirrors Chen et al.’s (2026) reading-context finding and extends it to the AOP domain. However, we anticipate that the magnitude of the autonomy → interest pathway may be stronger in the AOP context, where the choice of topic and presentation format has more immediate consequences for personal meaning-making than in standardized reading tasks.

### 3.2 Hypothesis cluster 2: Differential motivational pathways for social support

The most theoretically interesting—and most context-sensitive—propositions concern the pathways through which social support translates into SRL.

Theoretical Derivation for Social Support Pathways (H2a & H2b):

Drawing upon Social Cognitive Theory (Bandura, 1997; Pintrich, 2004), the interpersonal ecology of the classroom acts as a critical emotional and cognitive catalyst for self-regulation. Perceived teacher social support represents an authoritative relational anchor in the classroom. When teachers demonstrate high emotional care and responsiveness to student anxiety, they foster a safe climate that directly promotes adaptive motivational beliefs across multiple paths, reinforcing mastery goals, protecting self-efficacy, and elevating the perceived utility value of the learning process (Patrick et al., 2007; Roorda et al., 2017). Peer social support, however, must be structurally reconfigured within the AOP context due to the audience-evaluator duality that differentiates oral performance from reading or writing settings (He and Jin, 2026). In an academic presentation class, classmates do not remain passive, asynchronous co-learners; they constitute the immediate, live public face of evaluation and feedback (Liu and Hansen, 2002; Han and Wang, 2024). Consequently, peer support directly functions as a powerful socio-emotional buffer against severe performance anxiety and public face-threats (Wu and Schunn, 2023). An empathetic and non-threatening peer environment provides collective reassurance. This socio-emotional buffer bolsters the presenter’s self-efficacy through positive social persuasion and vicarious modeling (Bandura, 1997). Furthermore, constructive peer feedback validates the communicative success of the presentation, directly enhancing the speaker’s perceived utility value (He and Jin, 2026; Wu and Schunn, 2023).

*H2a:* Teacher social support exerts a broad, multi-path indirect effect on SRL strategy use through utility value, intrinsic interest, self-efficacy, and mastery goals. This proposition is grounded in the well-established literature documenting teacher support as a multidimensional motivational catalyst (Patrick et al., 2007; Roorda et al., 2017; Song et al., 2015; Wentzel, 1999, 2022). In the CHC context specifically, teachers’ authoritative status and the cultural emphasis on respect for educators amplify their motivational influence (Lau and Chen, 2013; Lee, 2021; Tao et al., 2022). We further anticipate that the strongest pathway will run through self-efficacy, given the AOP context’s elevated affective demands.

*H2b:* Peer social support exerts an indirect effect on SRL strategy use through reconfigured motivational pathways that reflect the audience-evaluator role of peers in the AOP context. Unlike in receptive-skill contexts—where peer support has been found to operate primarily through limited utility and self-efficacy pathways (Chen et al., 2026)—peers in the AOP context function simultaneously as collaborators, audience members, and feedback providers (He and Jin, 2026; Reinholz, 2016). We therefore propose that peer support in the AOP context exerts stronger effects through self-efficacy (via vicarious experience and audience-based reassurance) and through utility value (via the social validation that peer evaluation provides). This reconfiguration is a key theoretical contribution of the present framework.

### 3.3 Hypothesis cluster 3: Motivational beliefs as proximal drivers of SRL

*H3a:* All five motivational beliefs positively predict SRL strategy use in AOPs, with self-efficacy and intrinsic interest exhibiting the strongest associations. This proposition follows from Chen et al.’s (2026) finding in the reading context and extends it to AOPs, with the prediction that self-efficacy’s role is amplified in the AOP context owing to its proximity to anxiety regulation (Dao and Sato, 2021).

*H3b:* Associations between motivational beliefs and specific SRL strategy clusters are differentially weighted in the AOP context. Self-efficacy will show a particularly strong association with motivation-regulation strategies, while mastery goals will show a particularly strong association with metacognitive strategies. This differentiated pattern is theoretically novel and reflects the qualitative match between belief content and strategy function: self-efficacy directly supplies the affective resources for anxiety management, while mastery goals supply the long-term orientation needed for reflective monitoring and post-performance evaluation.

### 3.4 Contextual moderators: the Chinese higher education context

The above propositions, while grounded in general social-cognitive theory, are likely to be moderated by features of the Chinese higher education context.

First, the transition from examination-intensive secondary schooling to autonomy-oriented higher education generates within-cohort heterogeneity in motivational profiles (Chen et al., 2023; Zhong, 2006). Students who carry forward strong examination-oriented dispositions may show stronger performance-goal pathways, while students who successfully internalize academic autonomy may show stronger mastery-goal and intrinsic-interest pathways.

Second, the Confucian Heritage Culture orientation toward teacher authority (Lau and Chen, 2013; Lee, 2021) is likely to amplify the magnitude of teacher-support pathways relative to peer-support pathways, mirroring findings in CHC reading research (Chen et al., 2026; Patrick et al., 2007).

Third, the deeply entrenched “face culture” (mianzi) documented in East Asian educational psychology (Hu and Lam, 2010; King and McInerney, 2016) introduces unique social sensitivities. Empirical studies by Bharuthram and van Heerden (2023) and Lee (2021) demonstrate that public peer critique can induce defensive anxiety or communication withdrawal among Chinese students, thereby attenuating the direct benefits of peer support unless peer feedback protocols are explicitly structured to be non-threatening and constructive.

These moderating considerations do not refute the framework’s core propositions but rather sharpen their applicability and identify points where empirical investigation should examine boundary conditions.

### 3.5 Summary of testable propositions

Table 1 summarizes the propositions for ease of reference and to facilitate future empirical operationalization.

**Table 1:** Summary of core testable hypotheses, proposed pathways, foundational frameworks, and theoretical rationales.

Hypothesis	Proposed pathway	Foundational framework	Theoretical rationale (key citations)
H1a	Presentation task design & process-oriented teacher support → SRL (direct)	Cognitive scaffolding theory	Structured scaffolding affords strategy deployment regardless of motivation (Dignath and Büttner, 2018; Lau, 2019; Chen et al., 2026)
H1b	Study autonomy → intrinsic interest → SRL	Self-determination theory (Ryan and Deci, 2009)	Autonomy support nurtures intrinsic motivation (Ryan and Deci, 2009; Chen et al., 2026)
H2a	Teacher social support → multiple motivational beliefs → SRL (broad multi-path effect)	Social cognitive theory (Bandura, 1997)	Teacher support as multidimensional catalyst, amplified in CHC contexts (Roorda et al., 2017; Wentzel, 2022; Lau and Chen, 2013)
H2b	Peer social support → self-efficacy & utility value → SRL (reconfigured pathway)	Dual-role peer engagement model (He and Jin, 2026)	Peers as collaborators-audience-feedback providers in oral context (He and Jin, 2026; Reinholz, 2016; Wu and Schunn, 2023)
H3a	Five motivational beliefs → SRL (self-efficacy and interest strongest)	Expectancy-value framework (Eccles and Wigfield, 2002)	Direct motivational drivers of strategy use, extended to AOPs (Chen et al., 2023; Chen et al., 2026; Bandura, 1997)
H3b	Self-efficacy → motivation-regulation strategies (strong); mastery goals → metacognitive strategies (strong)	Achievement goal theory (Ames, 1992; Pintrich, 2004)	Qualitative match between belief content and strategy function (Dao and Sato, 2021; Ames, 1992; Pintrich, 2004)

## 4 Discussion

### 4.1 Reinterpreting recent evidence through the proposed framework

The framework introduced above offers a new lens through which to interpret several strands of recent empirical work. First, findings from the L2 reading SRL literature—particularly Chen et al.'s (2026) demonstration of differential motivational mediation for teacher and peer support—can be reinterpreted as instantiations of a broader principle that environmental dimensions operate through distinct motivational signatures. Our framework specifies how this principle is expected to manifest, with quantitative modifications, in the productive oral domain. Second, recent theoretical and empirical work on L2 peer feedback engagement (He and Jin, 2026; Wu and Schunn, 2023; Zhu and To, 2022) underscores the audience-evaluator duality of peers in oral contexts; our framework integrates this insight into a broader environmental theory by treating peer support not as a homogeneous construct but as a structurally distinct input compared to teacher support. Third, the literature on L2 oral anxiety and emotional engagement (Dao and Sato, 2021; Payant and Zuniga, 2022) can be theoretically connected to SRL by recognizing self-efficacy as the central motivational pathway linking affective experience to strategic behavior.

### 4.2 Theoretical contributions

The framework advances theory in three principal ways.

Extending SRL theory to productive oral skills. Whereas prior L2 SRL frameworks have substantiated the social-cognitive model primarily in reading and writing (Chen et al., 2023; Teng and Zhang, 2016; Zhang et al., 2014), the present framework articulates how the model operates—and how it requires adjustment—when applied to AOPs. The reconceptualized SRL strategy taxonomy (Section 2.1.3), with its expanded attention to motivation regulation, multimodal coordination, and in-performance monitoring, exemplifies this extension.

Opening the motivational black box. Although extant work has documented bivariate associations between classroom factors and SRL outcomes (Lau, 2012a, 2012b; Patrick et al., 2007), the specific motivational pathways through which environmental inputs translate into strategic behavior have not been adequately theorized in the AOP context. Our differentiated hypothesis structure (Section 3) provides a systematic account of these pathways and renders them empirically testable.

Bridging cognitive and social perspectives. Within L2 SRL research, cognitive perspectives (which emphasize individual strategy processing) and social perspectives (which emphasize interactional dynamics) have often operated in parallel rather than in dialogue (Aubrey, 2022). By positioning motivational beliefs as the mediator between socially situated environmental inputs and individually enacted strategic behavior, the framework provides a conceptual bridge between the two traditions. This integrative move parallels recent calls in the engagement literature to view learner engagement as a coupled, dynamic system rather than as a set of isolated dimensions (Aubrey, 2022).

### 4.3 The Chinese higher education context as theoretical scaffolding

The framework is explicitly situated in the Chinese college English context, and this situatedness is theoretically generative rather than parochial. The Chinese higher education context offers a productive case for theorizing several phenomena that may be less pronounced in other educational systems: the cohabitation of examination-oriented and academically oriented motivational orientations (Chen et al., 2023; Zhong, 2006); the cultural amplification of teacher authority (Lau and Chen, 2013; Lee, 2021); and the face-conscious negotiation of peer evaluation (Hu and Lam, 2010; King and McInerney, 2016). By foregrounding these features as moderators rather than as nuisance variables, the framework demonstrates how contextual specificity can enrich rather than constrain general theoretical claims (King and McInerney, 2016).

### 4.4 Pedagogical implications

Although the framework is primarily theoretical, it carries actionable implications for college English pedagogy.

First, AOP pedagogy should explicitly integrate SRL strategy instruction, including not only cognitive and metacognitive strategies but also motivation-regulation strategies—particularly those targeting anxiety management and self-encouragement (Spruce and Bol, 2015; Teng and Zhang, 2016). Such explicit, phased instruction is likely to be more effective than implicit modeling alone (Dignath and Veenman, 2021).

Second, teachers should consciously cultivate the affective dimension of their support, recognizing that teacher social support operates through multiple motivational pathways including self-efficacy and intrinsic interest (Roorda et al., 2017; Wentzel, 2022). Teacher professional development should accordingly emphasize social–emotional competencies alongside content and pedagogical knowledge (Chen et al., 2026).

Third, peer collaboration in the AOP context should be structured to leverage the audience-evaluator role of peers, for example through training in giving and receiving constructive peer feedback (He and Jin, 2026; Liu and Hansen, 2002), through cooperative rehearsal designs (Johnson and Johnson, 2018; Slavin, 1980), and through face-sensitive evaluation protocols (Bharuthram and van Heerden, 2023; Hu and Lam, 2010).

Fourth, task design should balance challenge with autonomy support, recognizing that autonomy operates indirectly through intrinsic interest. Token offers of “free choice” without accompanying interest cultivation are likely to yield limited SRL benefits.

### 4.5 Directions for future empirical inquiry: an operational research roadmap

To facilitate the empirical validation of the proposed theoretical framework and testable hypotheses, we delineate a concrete, four-part methodological roadmap for future researchers:

1. Operationalization and Instrument Adaptation. Future empirical studies should employ contextualized self-report scales adapted to the AOP setting:

Perceived Classroom Environment: Instructional practices (Presentation Task Design, Process-Oriented Support, Study Autonomy) can be operationalized by adapting Lau’s (2012a) Chinese SRL-based Reading Instruction Questionnaire (CSRIQ) and Ames's (1992) TARGET framework to oral presentation tasks (e.g., “Our teacher provides explicit guidelines on structuring oral presentation scripts”). Social support (Teacher and Peer Support) can be measured using adapted subscales from the Classroom Life Measure (Johnson et al., 1983; Patrick et al., 2007), specifically incorporating peer audience-evaluator items (He and Jin, 2026).

Motivational Beliefs: The five mediators can be assessed using adapted subscales from Pintrich’s (1991) Motivated Strategies for Learning Questionnaire (MSLQ), calibrated to public speaking self-efficacy, presentation utility value, intrinsic interest, and achievement goals (Ames, 1992; Eccles and Wigfield, 2002).

AOP SRL Strategies: Cognitive, metacognitive, and motivation-regulation strategies can be operationalized through an adaptation of Teng and Zhang's (2016) L2 SRL instrument, enriched with oral-specific items measuring anxiety management, gestural coordination, and live rehearsal monitoring.

2. Sampling Strategy and Target Population. We recommend multi-institution stratified cluster sampling targeting Chinese undergraduate non-English majors enrolled in compulsory College English or English for Academic Purposes (EAP) courses requiring graded oral presentations. Based on sample size guidelines for structural equation modeling with multiple latent constructs, a target sample size ranging between 400 and 600 participants (or minimally N≥400 ) is recommended to ensure model convergence and maintain adequate statistical power ( 1−β≥.80 at α=.05 ).
3. Statistical Testing Pipeline and Structural Equation Modeling. Hypotheses should be tested via a two-step Structural Equation Modeling (SEM) approach using statistical packages such as Mplus or R (lavaan):

Step 1 (Measurement Model): Conduct Confirmatory Factor Analysis (CFA) to verify construct validity, ensuring acceptable goodness-of-fit ( CFI≥.90 , TLI≥.90 , RMSEA≤.06 , SRMR≤.08 ).

Step 2 (Structural Model and Direct Effects): Estimate direct paths (H1a) using Maximum Likelihood estimation with robust standard errors (MLR).

Step 3 (Mediation Testing): Evaluate the specific indirect paths (H1b, H2a, H2b) using Bias-Corrected Bootstrapping (with 5,000 resamples) to generate 95% confidence intervals, confirming significant mediation when zero is excluded from the interval.

Step 4 (Contextual Moderation): Test the moderating role of cultural dispositions (e.g., face concern, exam orientation) via Multi-Group SEM or Latent Moderated Structural Equations (LMS).

4. Longitudinal and Experimental Designs. To overcome the limitations of cross-sectional survey designs and establish causal directionality, future inquiry should deploy three-wave longitudinal panel tracking ( $T_{1}$ : task introduction, $T_{2}$ : mid-preparation rehearsal, $T_{3}$ : post-presentation debriefing). Furthermore, quasi-experimental intervention designs (e.g., 2×2 factorial pretest-posttest control group designs) should be implemented to evaluate the explicit efficacy of teacher process-scaffolding modules and face-sensitive peer feedback training protocols in authentic higher education classrooms.

### 4.6 Boundary conditions and limitations

Three boundary conditions warrant explicit acknowledgment. First, the framework is calibrated to the Chinese college English context; its applicability to other linguistic, cultural, or educational settings requires careful adaptation and empirical re-examination (King and McInerney, 2016; Rovagnati et al., 2022). Second, the framework focuses on perceived classroom environment as reported by students; objective environmental features (e.g., teacher behaviors as coded by observers) may yield different patterns of association (Spruce and Bol, 2015). Third, the framework concerns proximal classroom influences and brackets more distal influences (e.g., institutional policies, parental expectations, broader sociocultural forces) that may also shape AOP-related SRL (Bronfenbrenner and Morris, 1998).

## 5 Conclusion

The development of self-regulated learning in college English academic oral presentations is not merely a matter of individual aptitude or linguistic competence; it is a process in which the classroom environment plays a formative role through specific motivational pathways. This paper has proposed a theoretical framework articulating how perceived instructional practices and social support translate into SRL strategy use in AOPs via differentiated motivational mediation. By extending the social-cognitive SRL paradigm into the productive oral domain, by theorizing the audience-evaluator reconfiguration of peer support, by specifying multi-path motivational mechanisms, and by foregrounding the Chinese higher education context, the framework offers a generative platform for both future empirical inquiry and pedagogical innovation. We hope it will catalyze research and practice oriented toward cultivating not just confident speakers, but reflective and self-regulating academic communicators.

## Data Availability

The original contributions presented in the study are included in the article/supplementary material, further inquiries can be directed to the corresponding author/s.
